# Simultaneous Bilateral Video-Assisted Thoracoscopic Surgery for the Treatment of Primary Spontaneous Pneumothorax

**DOI:** 10.1007/s12013-014-0393-7

**Published:** 2014-11-13

**Authors:** Xin Wang, Lei Wang, Huayong Wang, Hao Zhang

**Affiliations:** Department of Thoracic Surgery, Xuzhou Central Hospital, Xuzhou, 221009 Jiangsu China

**Keywords:** Primary spontaneous pneumothorax, Video-assisted thoracoscopic surgery, VATS

## Abstract

We determined the feasibility and clinical efficacy of simultaneous bilateral video-assisted thoracoscopic surgery (VATS) for treating primary spontaneous pneumothorax (PSP). We performed a retrospective analysis of the efficacy of simultaneous bilateral resection of pulmonary bullae using VATS in 21 PSP patients that were treated at our hospital from February 2010 to August 2013. We found bilateral bullae in all patients through the intraoperative exploration. Surgical procedures were successfully completed in all patients without conversion to thoracotomy. The mean time of surgery was 128.76 ± 13.82 min (range 100–150 min). Total amount of intraoperative bleeding was 80–200 ml. Total drainage of bilateral thoracic ducts was 200–500 ml at the 1st postoperative day with a mean drainage of 321.42 ± 82.66 ml. Bilateral thoracic ducts were removed 4–8 days postoperatively with a mean time of 4.7 days. The duration of postoperative hospitalization was 5–9 days with a mean duration of 7 days. No patient had serious complication(s) and all patients were discharged after full recovery. The patients were followed up for 6–18 months after the surgery and no relapse occurred. In conclusion, treating the unilateral PSP by simultaneous bilateral VATS is safe and effective. It reduces patients’ risk of second surgery and also minimizes patients’ suffering and costs incurred.

## Introduction

Spontaneous pneumothorax is a common disease treated by thoracic surgery. It refers to the rupture of lung parenchyma or visceral pleura due to non-foreign or interventional factors, leading to the accumulation of air in pleural cavity [1]. Primary spontaneous pneumothorax (PSP) is defined as a pneumothorax without the underlying lung disease, and it is usually caused by ruptured pleural blebs or bullae. It is more common in tall, slender, adolescent males, with a male/female ratio of about 5:1 [2]. The relatively higher incidence in males has been ascribed to factors, such as higher smoking rates, body habitus/physique, and different mechanical properties of the male lung [3].

The chest computed tomography (CT) has been commonly used to help understand the pathogenesis of PSP and plan out the management strategies. However, the recent development of video-assisted thoracoscopic surgery (VATS) has changed the management strategy profiles of PSP and thoracoscopic surgery has become a routine treatment procedure for this disease. Herein, we present a retrospective analysis of the efficacy of simultaneous bilateral resection by VATS of the pulmonary bullae in 21 patients with PSP that were treated at our hospital department during the time period from February 2010 to August 2013.

## Materials and Methods

### Patients

The patients were diagnosed with unilateral spontaneous pneumothorax (or hydropneumothorax) by chest X-ray/CT examination, and the contralateral pulmonary bullae were identified by chest CT examination. All patients fully understood their disease condition, agreed and requested for simultaneous bilateral VATS, and signed informed consent forms prior to surgery. The patients were excluded if they had a history of pneumothorax or previous surgery. Of the 21 patients, 20 patients were male and one patient was female, with a mean age of 22 (range 17–27) years. Fifteen patients had pneumothorax at first presentation and six patients had second or repeated pneumothorax; 12 cases were of the left pneumothorax and nine cases were of the right pneumothorax. Ten patients had preoperative pneumothorax with the lateral lung compression of >30 % and these individuals had received closed thoracic drainage, while the other 11 patients did not receive closed thoracic drainage preoperatively.

### Anesthesia

All patients received the double-lumen endotracheal intubation with combined intravenous anesthesia and intraoperative one-lung ventilation of the non-surgical side. Regarding patients who did not receive closed thoracic drainage, a wide-bore needle was inserted into the second intercostal space of midclavicular line on the affected side before anesthesia. Anesthesia was induced by using midazolam 5–10 mg; fentanyl 0.05–0.1 mg 2–3 min repeated injections; cisatracurium besylate 0.15 mg/kg; etomidate 150–300 μg/kg; and maintained by propofol 4–12 mg/kg/h; and rui fentanyl 0.1–2 μg/kg/min.

### Surgical Methods

**Fig. 1:**
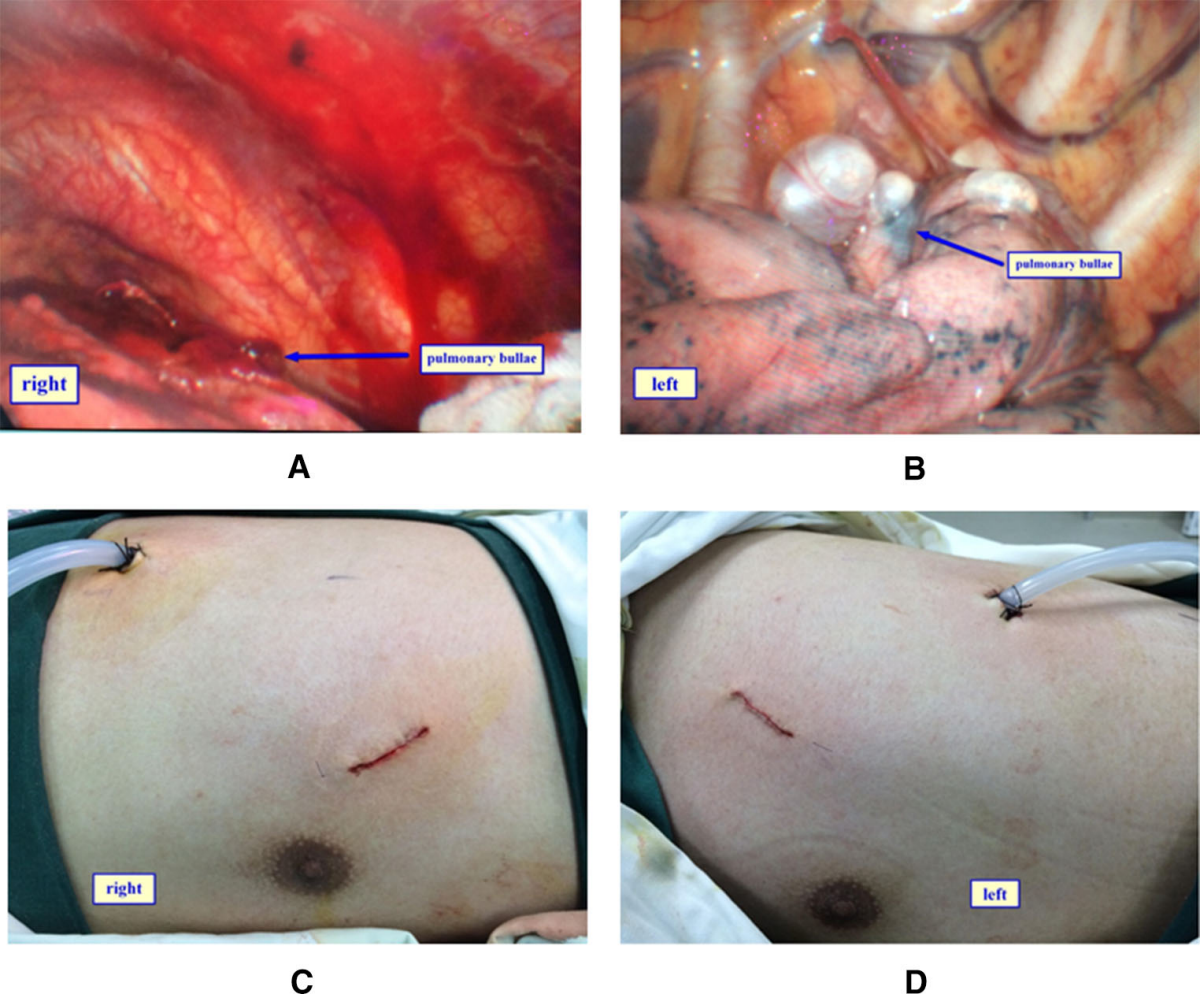
Exposing the *right* (**a**) and the *left* (**b**) pulmonary bullae is shown in a representative patient of primary spontaneous pneumothorax. Following resection by simultaneous bilateral video-assisted thoracoscopic (VAT) surgery, closure and drainage of the *right* (**c**) and the *left* (**d**) chest wall are also shown

**Fig. 2:**
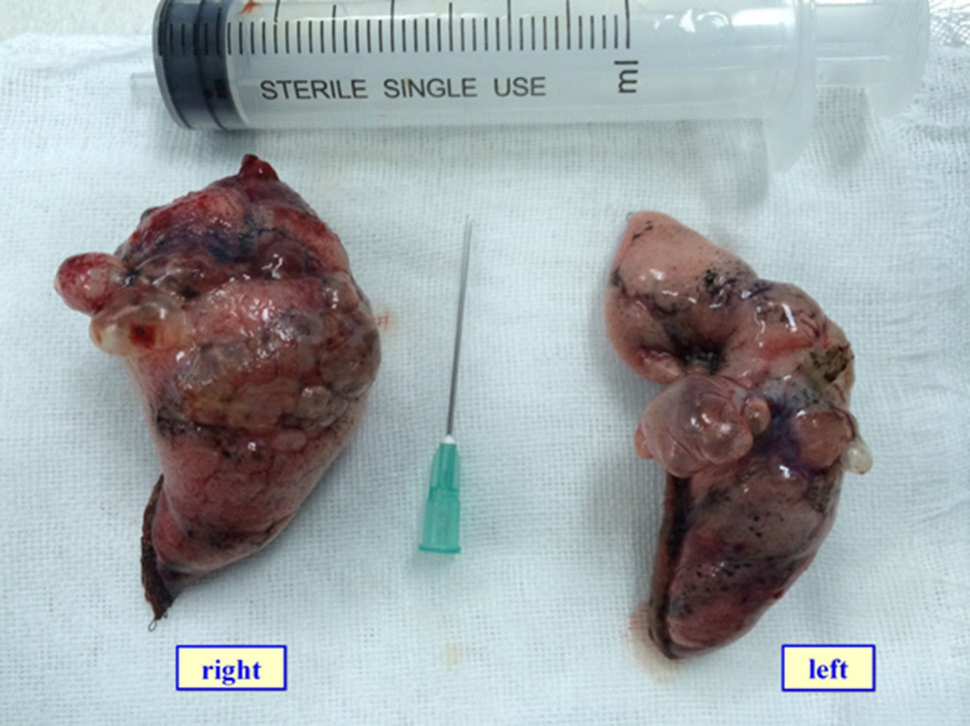
The *right* and the *left* side bullae resected from the above patient are shown

A 30° and 10 mm thoracoscope and optical source, endoscopic linear stapler, and staple cartridge were used in this VAT surgery. The patient was recumbent in the lateral position with the upper arms extended and fixed on the hand support. First, unilateral pneumothorax surgery was performed and then the patient was flipped over to other side to perform the contralateral surgery. The VAT surgery was performed with a 1.5 cm incision, made in the 7th or 8th intercostal space; the mid-axillary line was the observational pole. A thoracoscopic puncture cannula was inserted and thoracoscope was indwelled for exploration. Based on the preliminary exploration results, an incision of 1.5–2.0 cm was made at the outer margin in the 3rd or 4th intercostal ectopectoralis as the operation hole where the incision protective sleeve was indwelled. A sponge-holding forceps was placed in the operation hole to clamp the lung tissue and determine the exact location of the pulmonary bulla. If pleural adhesion was found, it was separated and incised using electric hook or ultrasonic knife. In case of thick pleural adhesions, the small vessels were occluded and severed with titanium clip or Hem-o-lok. Pulmonary bulla was lifted with sponge-holding forceps, basal part was clamped and the endoscopic linear stapler was used to remove the pulmonary bulla or pulmonary air leakage tissue. Sterile warm physiological saline was infused into the pleural cavity before the end of surgery and pulmonary dilatation was performed. After ensuring that no pulmonary bulla was ignored or no pulmonary air leaked, the visceral pleura and parietal pleura were rubbed clean with gauze, thoracoscope was moved to the operation pole before closure, and an intrathoracic drain was placed in the observation pole under thoracoscopy to ensure full pulmonary dilatation. The operation pole was sutured with needle-free stapler or intradermal suture. Exposing of the right (Fig. 1a) and left (Fig. 1b) pulmonary bullae as well as closure/drainage of the right (Fig. 1c) and left side walls are shown in a representative case. The right and left side resected bullae in this patient are shown in Fig. 2.

## Results

### Location, Number, and Size of Pulmonary Bullae

Bilateral pulmonary bullae were found in all patients at intraoperative exploration, including 18 cases (85.7 %) of pulmonary bullae in the apex of superior lobe and 3 cases (14.3 %) in the middle or inferior lobe. Seventeen cases (80.9 %) were of multiple pulmonary bullae and 4 cases (19.1 %) were of single pulmonary bullae; in five cases (23.8 %), the maximum diameter of pulmonary bullae was ≥1 cm and in 16 cases (76.2 %), the maximum diameter of pulmonary bullae was <1 cm. The data are summarized in Table 1. The presence of pulmonary bullae was confirmed pathologically in all bilateral lung tissues removed.

**Table 1:** Location, number, and size of pulmonary bullae

Location			Type		Diameter	
Superior lobe	Middle lobe	Inferior lobe	Multiple pulmonary bullae	Single pulmonary bulla	≥1 cm	1 cm
18 cases	1 case	2 cases	17 cases	4 cases	5 cases	16 cases

### Patients’ Clinico-Surgical Data

All patients successfully completed VATS surgery without conversion to thoracotomy. As outlined in Table 2, the mean duration of surgery was 128.76 ± 13.82 (range 100–150) min. Mean intraoperative bleeding was 124.28 ± 32.18 (range 80–200) ml. The total drainage of bilateral thoracic ducts at first day postoperatively was 200–500 ml, with a mean drainage of 321.42 ± 82.66 ml. Bilateral thoracic ducts were removed at 4–8 days postoperatively, with a mean removal time of 4.7 days. The duration of postoperative hospitalization was 5–9 days, with a mean duration of 7 days. None of the patients had serious complication(s) and all patients were discharged with recovery. The patients were followed up for 6–18 months after discharge and no relapse occurred.

**Table 2:** Patients’ clinical data

Patient number	Duration of surgery (min)	Total intraoperative bleeding (ml)	Bilateral thoracic ducts total drainage at 1st day postoperatively (ml)	Bilateral thoracic ducts duration (days)	Duration of postoperative hospitalization (days)
1	120	100	200	5	6
2	150	100	550	4	5
3	140	80	250	5	9
4	125	120	300	5	8
5	130	100	300	4	7
6	139	160	400	6	5
7	140	160	250	4	8
8	150	150	260	5	6
9	120	150	450	5	7
10	130	120	350	8	7
11	120	100	300	5	7
12	100	100	260	5	8
13	120	100	290	4	6
14	110	120	300	5	9
15	150	200	240	4	6
16	110	150	280	4	5
17	120	180	260	4	9
18	130	120	400	5	7
19	125	100	360	4	8
20	140	100	380	4	7
21	135	100	370	4	9
Mean ± SE	128.76 ± 13.82	124.28 ± 32.18	321.43 ± 82.66	4.71 ± 0.96	7.09 ± 1.34

## Discussion

The cause of PSP is the rupture of subpleural bullae [4]. The formation of pulmonary bullae in bilateral pulmonary apex is most commonly found in the middle-aged or young patients. Although many patients herein operated presented with unilateral pneumothorax, the contralateral lung had a history of pneumothorax or was found to have pulmonary bullae as also described previously [5]. In order to prevent contralateral pneumothorax, these patients needed to have simultaneous bilateral bulla resection. Previously, simultaneous bilateral surgery required bilateral thoracotomy or median sternotomy that involved high risks with regard to surgery and anesthesia, large trauma, long time of thoracotomy and closure, more intraoperative bleeding, and significant postoperative pain. Consequently, it was difficult for patients to undergo the massive surgical procedures involved. With the development of VATS technology, it has now become feasible to perform simultaneous bilateral bulla resection. VATS pulmonary bulla resection has substantial advantages, such as minimally invasive procedure, less intraoperative bleeding, short surgical time, and fast postoperative recovery [6, 7]. Simultaneous bilateral pulmonary bulla resection performed for the unilateral pneumothorax has dual benefit i.e. curing the unilateral pneumothorax and preventing the contralateral pneumothorax [8, 9]. Thus, this surgical procedure can exempt patients from the risk and suffering of another surgery later on; as well as reduce the medical costs and hospitalization time, and lessen burden on already strained healthcare system.

The main objective of simultaneous bilateral VATS was to prevent the recurrence of pneumothorax, as the left over pulmonary bulla(e) during surgery were reported to cause postoperative relapse of pneumothorax [10]. It remains very important to confirm the location, number, and size of pulmonary bullae at intraoperative exploration. Jianxing reported that conventional chest X-ray could not obtain full pulmonary information from each section and location, while the chest CT had identified pulmonary bullae of sizes ≥0.8 cm [11]. All patients should have chest X-ray before surgery, or a high-resolution thin-section CT scan if required, to identify the pulmonary bulla(e). At the end of surgery, we infused warm physiological saline and dilated the lung to examine whether there was air leakage or ignored pulmonary bulla(e); and at this time, we also switched the thoracoscope from the observation pole to the operation pole in order to have thorough observation from two angles. The pneumothorax relapse can also be caused by insufficient adhesion in the pleural cavity [12]. Therefore, we rubbed the visceral pleura and parietal pleura with gauze ball and 50 % glucose solution before closure in order to eliminate the pleural cavity gap and reduce the chances of pneumothorax recurrence as recommended [13].

With regard to surgical position of patient, some investigators prefer the lateral position and later change to the contralateral position after the unilateral surgery [14, 15], while others propose the dorsal position for surgical efficacy [16]. In most PSP patients, the pulmonary bullae were found in the apex of superior lobe [17]; hence, the dorsal position might not be suitable for exploring lesions in the posterior segment of the superior lobe and the dorsal segment of the inferior lobe. By flipping position and performing surgery after the first surgery in the lateral position, we needed to examine whether the double-lumen tubes were moved due to position change and we found that using fiberoptic bronchoscope was quite helpful as was suggested previously [8]. Of note, we did try to use dorsal position in a few cases of bilateral surgery as flipping patient position required operation site re-preparation (disinfection, draping, etc.); however, the operation flexibility of dorsal position was not enough and it was not a suitable position for difficult pulmonary bulla resection. For patients whose lung function may be compromised in the lateral recumbency or whose lesions are easily accessible in the dorsal position, it may be more advantageous to use the dorsal position.

Importantly, the duration of simultaneous bilateral surgery is longer than unilateral surgery and the need of one-lung ventilation is also stricter; therefore, the patient’s cardio-pulmonary function should be assessed before surgery in order to determine suitability for simultaneous bilateral surgery; otherwise, thoracotomy or unilateral surgery may be considered. Also, maintaining the intraoperative stability of respiratory and circulatory systems is pivotal to the success of surgery. It is important to ensure the correct location of double-lumen tubes during anesthesia as well as after position change; a fiberoptic bronchoscope may be used for examination if necessary. It is essential to remove the secretions timely from the respiratory tract during surgery and thus keep the respiratory tract unobstructed. Besides, in order to prevent tension pneumothorax on the pneumothorax side due to mechanical ventilation, we performed the surgery on the pneumothorax side first; however, the intraoperative ventilation increased the possibility of pulmonary bulla rupture on the non-surgical side. In addition, it is crucial to monitor the oxygen saturation, change of airway pressure, and/or occurrence of tension pneumothorax on the non-surgical side. If the oxygen saturation decreases, airway pressure increases, and breathing sounds are weakened on the non-surgical side, and tension pneumothorax may occur. Given these circumstances, it was recommended to assess the situation timely and perform the desired treatment [18]. Simultaneous bilateral VATS would not result in pulmonary compensation on the healthy side; therefore, we should prevent repeated operations and repeated clamping and squeezing of the lung tissue. Moreover, we should perform slow and fractionated pulmonary dilatation postoperatively in order to prevent the rapid dilatation and over dilatation.

In summary, simultaneous bilateral VATS can successfully cure the unilateral pneumothorax and prevent the contralateral pneumothorax in PSP patients. It is a safe and effective as well as minimally invasive procedure. It reduces patient’s risks of second anesthesia/surgery and is both time- and cost-effective. Therefore, it can be used as a treatment of choice for PSP patients.
